# Recent developments in the use of gold and silver nanoparticles in biomedicine

**DOI:** 10.1002/wnan.1817

**Published:** 2022-07-01

**Authors:** George Pasparakis

**Affiliations:** ^1^ Department of Chemical Engineering University of Patras Patras Greece

**Keywords:** COVID‐19, diagnostics, gold nanoparticles, nanomedicine, silver nanoparticles

## Abstract

Gold and silver nanoparticles (NPs) are widely used in the biomedical research both in the therapeutic and the sensing/diagnostics fronts. Both metals share some common optical properties with surface plasmon resonance being the most widely exploited property in therapeutics and diagnostics. Au NPs exhibit excellent light‐to‐heat conversion efficiencies and hence have found applications primarily in precision oncology, while Ag NPs have excellent antibacterial properties which can be harnessed in biomaterials' design. Both metals constitute excellent biosensing platforms owing to their plasmonic properties and are now routinely used in various optical platforms. The utilization of Au and Ag NPs in the COVID‐19 pandemic was rapidly expanded mostly in biosensing and point‐of‐care platforms and to some extent in therapeutics. In this review article, the main physicochemical properties of Au and Ag NPs are discussed with selective examples from the recent literature.

This article is categorized under:

Therapeutic Approaches and Drug Discovery > Nanomedicine for Oncologic Disease
Diagnostic Tools > In Vitro Nanoparticle‐Based Sensing
Nanotechnology Approaches to Biology > Nanoscale Systems in Biology

## Graphical abstract

Properties and Applications of Gold and Silver Nanoparticles in Therapeutics, Diagnostics and Biosensing Applications.

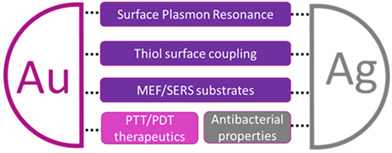

## 1 INTRODUCTION

**FIGURE 1:**
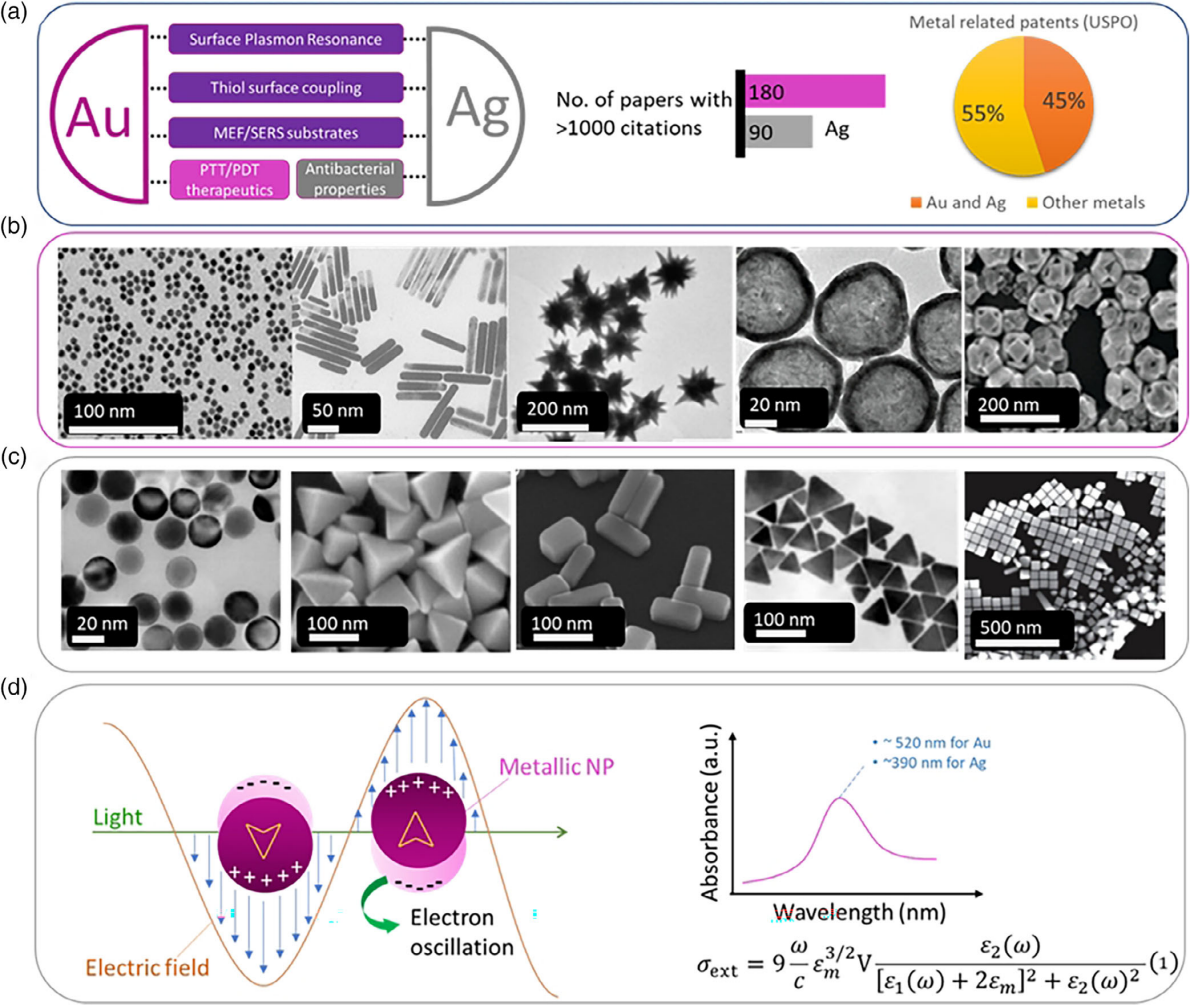
(a) Key common and distinct properties of Au and Ag nanoparticles (NPs) in the biomedical field along with representative metrics of the size of the current research and patent landscape; (b,c) depict spherical and anisotropic geometries of Au (spheres, rods, stars, shells, and cages) and Ag (spheres, pyramids, triangles, and cubes) NPs, respectively, that have been explored for biomedical applications, and (d) interaction of a light wave with a spherical metallic NP causing oscillation of the electron cloud on the surface of the NP, giving characteristic absorbance spectra for Au and Ag, which were first described by Mie equations. Copyrights by (b,c) Springer Nature (Cobley et al., 2009; Raliya et al., 2017), American Chemical Society (Adams et al., 2014; Skrabalak et al., 2008; Y. Sun et al., 2003; Wiley et al., 2006, 2007; Y.‐Y. Yu et al., 1997; S. Zhou et al., 2016), and Institute of Physics (Navarro et al., 2012)

Gold and silver nanoparticles (NPs) are arguably the most widely used metals in the field of biomedical sciences with ever increasing applications in research areas such as controlled drug delivery systems, precision therapeutics, sensors, and diagnostics. The research landscape of this research field has continued to expand in the last 5–10 years with increasing number of papers for both metals, while the number of research papers with more than 1000 citations as of February 2022 has now reached 270 for Au and Ag combined, testament of the relative maturity stage of the field (Figure 1a). Proportionally, out of the total number of patents related to metallic NPs in general that have been filed at the US Patent and Trademark Office, 45% are directly related to Au or Ag NPs, reflecting the potential future outlook of the field for real‐world applications (data mined at February 2022, Figure 1a). Finally, there are 49 clinical studies (completed or ongoing, data retrieved from ClinicalTrials.gov) related to precision oncology and inflammation, as well as antibacterial therapeutics and hence it is reasonable to expect that various commercial products utilizing the properties of Au/Ag NPs will emerge in the near future.

Given the vast bοdy of literature and the relatively large number of excellent review articles that cover virtually all aspects of Au and Ag NPs and their biomedical applications, this review focuses on providing the reader with key recent examples ca. of the last 5–7 years that cover (occasionally niche but) emerging areas in the fields of therapeutics, medical imaging, and (bio‐)sensors with interest in (point‐of‐care) diagnostics. Key examples of bimetallic Au and Ag NPs that exert combinational properties are also discussed. Of great interest are examples of image‐guided drug delivery applications, which fully harness the unique optical and chemical properties of these NPs in the biomedical context and especially in precision medicine.

Both Au and Ag NPs have been extensively studied as carriers for controlled drug delivery applications and share some common properties which render them appealing for this research area; namely, they can be synthesized relatively easily, at various sizes and shapes, their surface can be functionalized with various moieties, such as polymers, peptides, targeting ligands, imaging probes, and so on, and they are generally regarded as safe materials for certain in vivo bioapplications (Figure 1b,c). However, Au NPs constitute a far more versatile nanoplatform, especially for in vivo applications, mainly for two reasons: (1) They have oxide‐free surfaces and are considered more bioinert compared to Ag NPs and (2) Ag NPs can pose toxicity issues due to Ag^+^ release. The latter reason actually explains the intrinsic antibacterial properties via Ag^+^ release which induces disruption of the negatively charged bacterial cell wall, inhibition of DNA replication and blocking of respiratory enzyme activity, and hence their uses are mostly directed toward antibacterial therapeutics (Figure 1a) (Chernousova & Epple, 2013; Wijnhoven et al., 2009). A common feature of Au and Ag nanostructures is that they constitute excellent substrates to form self‐assembled monolayers (Ulman, 1996); for example, alkanethiol molecules can be self‐assembled in a closely packed manner on the surface of Au and Ag surfaces by the formation of Au—S and Ag—S bonds (Laibinis et al., 1991), respectively; the possibility to alter the macroscopic properties of surfaces by addition of a single‐molecule layer via chemisorption was initially explored in soft lithography (Delamarche et al., 1996; Xia & Whitesides, 1998) but was quickly diffused to other fields of research, such as the decoration of spherical particles with thiol‐rich biomolecules (including proteins and nucleic acids), (semi‐)telechelic polymers, drug molecules as well as fluorophores and imaging tags.

This review article by no means seeks to discuss all literature (probably impossible!) but to give an overview of the most important properties of Au and Ag NPs in the context of the biomedical field by discussing examples from the recent literature, where possible. In the context of therapeutics, the two categories are described in different sections since their therapeutic applications are derived from different properties (i.e., Ag NPs exhibit intrinsic antibacterial activity while Au NPs do not); conversely, for biosensing applications the two classes of NPs are grouped together as they share common properties and are often combined. Examples from the recently emerging field of bimetallic Au–Ag NPs for therapeutics and diagnostics are also analyzed. Finally, recent examples on how these remarkable nanomaterials have been explored in the SARS‐CoV‐2 pandemic era are given.

## 2 AU NPs: PHOTOTHERMAL, PHOTODYNAMIC, AND IMAGE‐GUIDED THERAPEUTICS

Noble metal NPs (i.e. Au, Ag, and Cu) have a so‐called surface plasmon resonance (SPR) caused by the oscillation of the conduction electrons on the surface of the particles upon light irradiation (Figure 1c) (Kelly et al., 2002). The research of colloidal dispersion of metallic NPs dates back to Faraday (1857) and the first systematic report on the occurrence of the SPR was first reported by Wood (1902). Soon after, Mie successfully reported a solution of Maxwell's equations that described sufficiently the extinction spectra of spherical particles (Mie, 1908). For NPs with radius r significantly smaller than the interacting light wavelength *λ* (2*r* < <*λ*), the extinction cross‐section can be described by Equation (1) (Figure 1d), where *V* is the particle volume, ω is the angular frequency of the exciting light, *c* is the speed of light and *ε*_*m*_ and *ε*(*ω*) *= ε*_1_ *+ iε*_2_(*ω*) are the dielectric functions of the surrounding medium and the material itself, respectively (Link & El‐Sayed, 2010). SPR is a nanoscale property of metallic NPs with sizes well below the wavelength of irradiation (but no less than 2 nm in diameter where quantum size effects are observed) and is not seen in bulk metals. The SPR is responsible for the characteristic bright color of metallic NPs. Indicatively, 10 nm Au NPs have a SPR characteristic band at ca. 520 nm while for Ag this occurs at ca. 390 nm (Figure 1d); typically, spherical Au and Ag NPs have a red and yellow color in colloidal solutions, respectively. The SPR can be red‐shifted to a limited range by tuning the size of the particles, however, for biomedical applications where more red‐shifted SPR bands are required (typically in the red and near‐infrared [NIR] regimes [650–900 nm], where human tissue and biological fluids have minimum absorption) the use of nonspherical particle geometries are preferred (Jain et al., 2008). For example, rod‐shaped NPs have two distinct resonances, one due for the short axis and another one due to the long axis; the length‐to‐width ratio is a critical parameter to tune the red‐shifting and the overall oscillation strength of the SPR.

The possibility to tune the plasmon resonance of Au NPs toward and beyond the NIR regime of the electromagnetic spectrum in combination of the aforementioned properties directed researchers to explore Au as a nanoplatform for photothermal therapeutics. More specifically, Au NPs in the form of nanoshells, nanorods, nanostars, nanocubes, and nanotriangles are now synthetically accessible by various synthetic routes allowing their biomedical use. The optical properties of these NPs are mainly controlled by their shape (Grzelczak et al., 2008; Liz‐Marzán, 2005). For example, as previously mentioned, the surface plasmon of gold nanorods is split into two distinct modes proportional to the aspect ratio (long/short axes ratio) showing a weak transverse resonance at ca. 520 nm and a more intense longitudinal SPR ranging from ca. 650 to 1300 nm (Pérez‐Juste et al., 2005). Similar hybridization of the plasmons is also seen in Au nanostars derived by the core/tips topology which results in the appearance of a tuneable plasmon band in the red/NIR regime (Barbosa et al., 2010). Au nanoshells show strong red/NIR shifting of their plasmon resonance depending on the particle geometry and shell thickness due to the hybridization of the plasmons on the inner and outer surface of their shell (X. Huang & El‐Sayed, 2010; Prodan et al., 2003). More recently developed Au nanocages constitute another geometry and resemble the structure of Au nanoshells in that they can also have an inner cavity with thin, porous cubic wall geometry (Chen et al., 2007).

### 2.1 Some interesting Au NP nanoformulations

**FIGURE 2:**
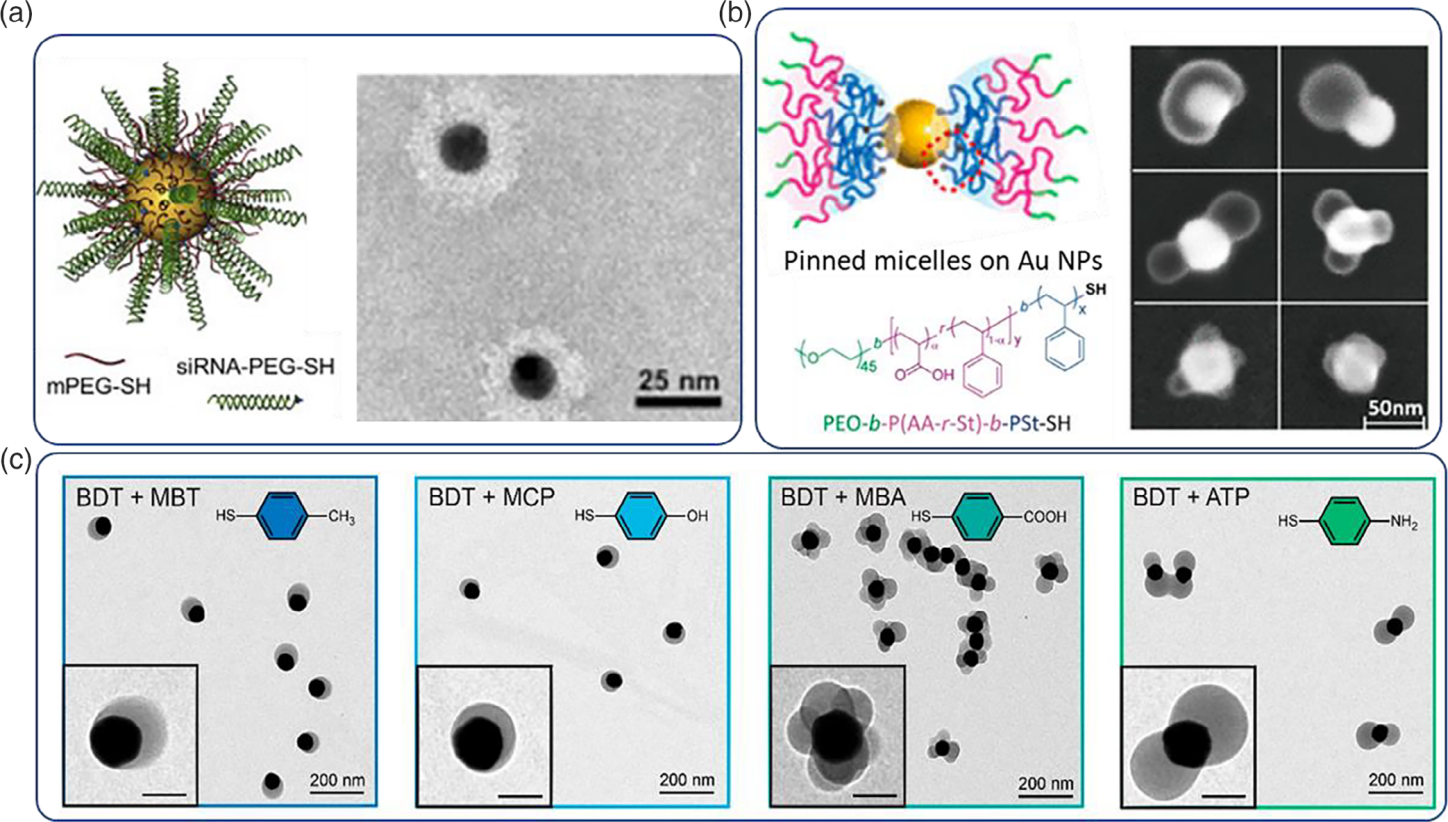
Schematic and transmission electron microscopy (TEM) images of (a) a spherical nucleic acid (SNA) with Au NP core and a densely packed siRNA and poly(ethylene glycol) shell, (b) poly(ethylene oxide)‐b‐poly(acrylic acid‐r‐styrene)‐b‐poly(styrene)‐SH triblock copolymer self‐assembly of thiol‐capped polymer chains on the surface of Au NPs with patchy anisotropic geometries, and (c) facile anisotropic geometries on the surface of Au NPs by self‐assembly and polymerization of small ionizable ligands (namely, benzene‐1,4‐dithiol [BDT] mixed with other thiol ligands depicted in the inlets) controlled by their respective stoichiometries. Copyrights by (a) Elsevier (Melamed et al., 2018) and (b) American Chemical Society (Y. Yang, Lina, et al., 2021; J. Zhou et al., 2021)

In the vast majority of studies Au NPs are passivated with protein‐repellent moieties (i.e., synthetic polymers, Salmaso et al., 2009; biomolecules, Bolaños et al., 2019; or charge‐bearing ligands, DeVries et al., 2007) to increase their blood plasma circulation times and mitigate the issue of fast opsonization and elimination (Amina & Guo, 2020). Thiol‐terminated polyethylene glycol (PEG) is the polymer of choice for Au NP passivation (although there exist plentiful alternatives; Barz et al., 2011; Thi et al., 2020), which can be combined with additional thiol‐functionalized targeting ligands, imaging probes, or even drug/biologic molecules to construct fully functional targeted nanoconstructs (Jokerst et al., 2011). Spherical nucleic acids (SNAs) constitute another interesting type of nanostructure, which are composed of Au NP cores with densely functionalized nucleic acid molecules (Figure 2a) (Mokhtarzadeh et al., 2019). SNAs confer physicochemical properties which differ from their structural components; for example, they are resistant to nuclease degradation, they exert cooperative binding events, they are capable to transfect multiple cell lines with high transfection efficiency, and they are highly versatile to accommodate single or double‐stranded nucleic acids (Cutler et al., 2012). Deng et al. recently improved the original synthetic route to form SNAs with the use of a simple sequential dehydration/rehydration protocol with butanol (Hao et al., 2021). The proposed method results in highly dense SNAs in a few seconds; such simple preparation routes can potentially render functional Au NPs even more appealing for applications in healthcare technologies such as diagnostics, therapeutics and nanosensors design. More precise nanoconfinement and ultimately control on the topology at the nanoscale was possible with the employment of controlled polymerization methods that allow for superior control of the macromolecular architecture (H.‐H. Jeong et al., 2019; P. P. P. Kumar & Lim, 2021). Fine control over the surface functionalization topology can be achieved with the use of well‐defined block copolymers or by in situ polymerization and self‐assembly of small ligands in presence of Au NPs which can result in hierarchical and anisotropic geometries with excellent fidelity (Y. Yang, Lina, et al., 2021; J. Zhou et al., 2021) (Figure 2b,c). Au NPs exert excellent light‐to‐heat conversion rates enabling the laser‐directed treatment of cancer tumors, by direct photothermal ablation or via apoptotic events by mild hyperthermia (Amendoeira et al., 2020). West et al. were the first to deploy preclinical studies on the photothermal treatment of solid tumors with the use of gold nanoshells (silica core, gold shell, 130 nm of total diameter) as photothermal antennae (Rastinehad et al., 2019). It was shown that laser irradiated (820 nm, 4 W/cm^2^) Au nanoshells could induce lethal local hyperthermia at tumor sites. Encouraged by the results, the same group recently reported on the photothermal ablation of prostate tumors in human subjects in the first clinical pivot study utilizing Au nanoshells (Rastinehad et al., 2019). Interestingly, the nanoshells were administered by intravenous infusion and were found to accumulate at tumor sites at concentrations of 3.5 times higher compared to normal tissue; an impressive 87.5% of lesions were negative for tumor in the ablated areas 12 months posttreatment. The efficient light‐to‐heat conversion properties of Au nanoshells were exploited by Day et al. to release miRNA to triple negative cancer cells (Dang et al., 2021); miRNA was immobilized by Au‐s bonds which could be cleaved by pulsed laser irradiation; photothermal de‐hybridization could also induce the release of RNA strands if they are mounted in the form of double strands due to the local heat confinement during irradiation. It was found that pulsed laser irradiation was more efficient in the release events and was an effective means to deliver the RNA cargo in vitro (i.e., irradiating for 10 min with 0.1 W/cm^2^, ca. 30% and 16% of nanoshell‐loaded miRNA duplexes were released with pulsed and continuous wave laser source, respectively). Pulsed laser irradiation results in confined Au—S bond dissociation with negligible local heat buildup compared to continuous wave laser sources that induce DNA dehybridization as the main mechanism of nucleic acid release from plasmonic NPs (Goodman et al., 2017). A unique feature of Au NPs is that they allow for the development of combinational photo‐ and chemo‐therapeutics in a highly compartmentalized manner. Recently, we developed a polymer‐coated Au nanoshell nanoformulation which could exert confined hyperthermia and also deliver gemcitabine against pancreatic cancer cells (Emamzadeh & Pasparakis, 2021). We found that the native cytotoxicity of the drug could be shattered and completely restored and even augmented upon photothermal stimulation. This approach implies that it may be possible to reduce the systemically required drug dose for effective therapy and vice versa it may be possible to increase the Au NP‐loaded dose above the toxicity limits and release it only at the sites of irradiation enabling for highly aggressive therapeutic modalities which are not possible to achieve with non‐nanoformulated approaches.

**FIGURE 3:**
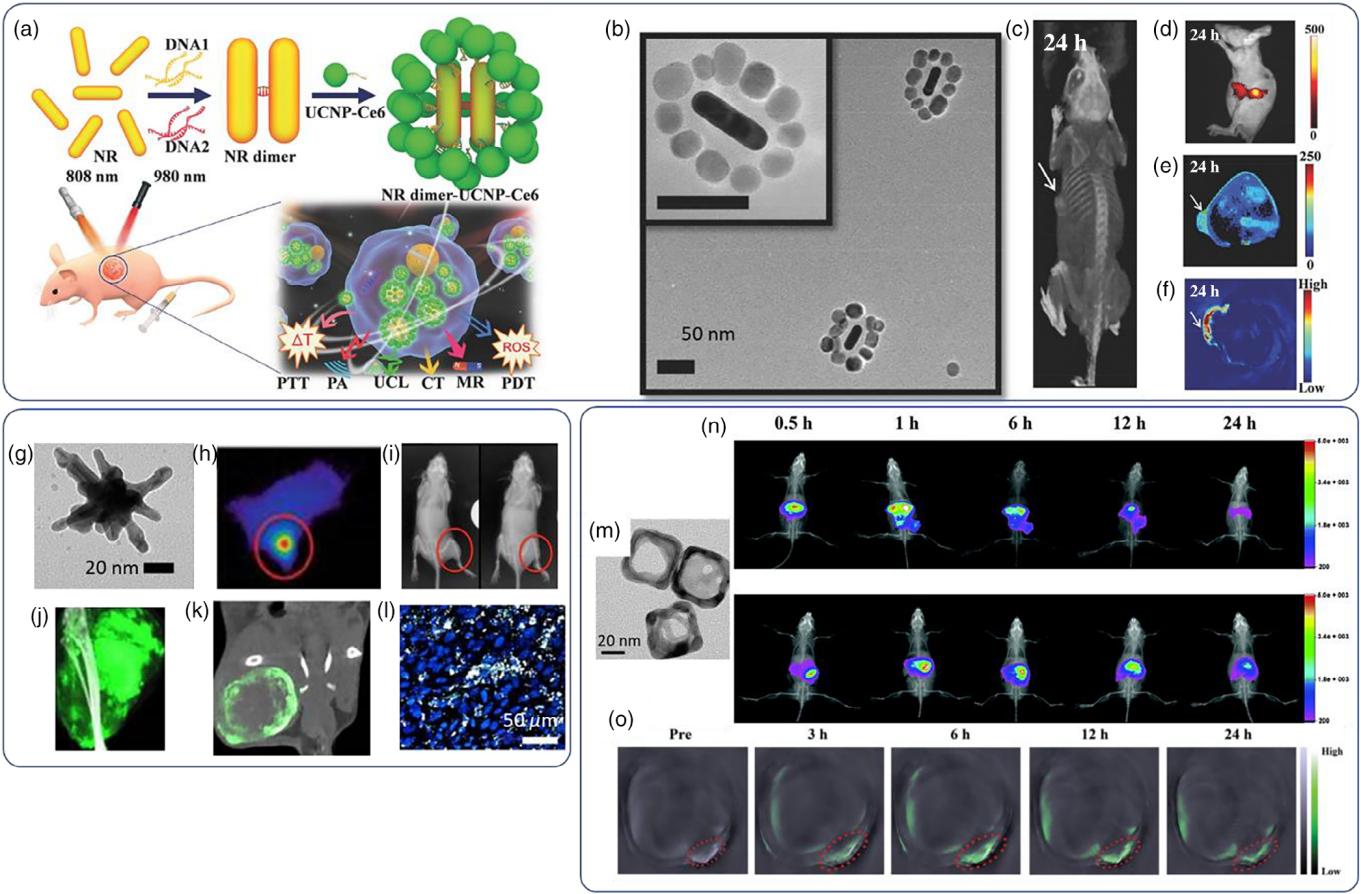
(a) Schematic of DNA‐glued nanorod (NR) dimers further self‐assembled with upconverting nanoparticles (NPs) with Ce6 photosensitizer for multimodal image‐guided combinational phototherapy, (b) representative TEM image of the nanoassemblies, (c) CT imaging of HeLa‐tumor bearing mice 24 h post i.v. injection of the nanoassemblies along with upconversion luminescence in (d) T1‐MR in (e) and photoacoustic imaging in (f). TEM image of a 30‐nm‐sized Au nanostar in (g), near‐infrared imaging of mouse surface temperature during photothermal treatment of primary sarcomas after intravenous injection with Au nanostars with characteristic temperature gradient increase (yellow–orange–red hues) in (h), X‐ray images of mice before and 3 days after photothermal treatment showing significant tumor reduction in (i), CT images of hind leg in primary sarcomas 24‐h postinjection with NPs showing increased NP accumulation at the tumor site (green signal) in (j), coronal CT‐slice of the tumor site 72‐h postinjection showing peripheral NP accumulation at the tumor site in (k), and two‐photon luminescence of Au nanostars with characteristic white signal in (l). TEM images of Au nanocages in (m), photoacoustic (PAT) images showing gradual biodistribution of Au nanocages in normal (top) and orthotopic mouse models (BEL‐7402, bottom) at time intervals up to 24 h in (n), tumor gradual tumor accumulation (green signal) of Au nanocages at the tumor site up to 24 h in (o). Copyrights by (a–f) Wiley (Langer et al., 2020); (g–l) Ivyspring (Y. Liu et al., 2015); and (m–o) Royal Society of Chemistry (Bao et al., 2017)

Recently, the photothermal properties of Au NPs were combined with other therapeutic modalities, such as photodynamic therapy (PDT) for augmented efficacy. Huang et al. used an efficient strategy to confine Au nanorod dimers with porphyrin‐functionalized upconverting NPs that generate cytotoxic singlet oxygen (^1^O_2_) by NIR irradiation (980 nm) (Figure 3a–f) (M. Sun, Xu, et al., 2016). The ensembles could be synthesized with high fidelity owing to the use of DNA hybridization strategies that selectively “glued” all components in a highly hierarchical manner. The reported formulation exhibits simultaneous photothermal (808 nm) and photodynamic activity against HeLa tumors which were completely eliminated 15 days posttreatment. It is noteworthy that this formulation served as an excellent imaging modality as it was possible to trace the tumors by PAT, computerized tomography (CT) X‐ray, and magnetic resonance (MR) imaging methods due to the passive accumulation of the NPs by the enhanced permeation and retention (EPR) effect (Rosenblum et al., 2018; Subhan et al., 2021). In a similar context, Badea, Vo‐Dinh and co‐workers studied systematically the photothermal and imaging properties of Au nanostars (Y. Liu et al., 2015). It was found that nanostars with a diameter of 30 nm could accumulate and penetrate sarcoma tumors much more efficiently than their 60‐nm counterparts and more interestingly, they had very high heat conversion efficiency, up to 94% allowing for efficient photothermal tumor treatment at relatively low laser doses (0.7 W/cm^2^) (Figure 3g–l); it was demonstrated that Au nanostars could be used as in vivo imaging probes via CT (by facile radiolabeling with I_131_), surface‐enhanced Raman scattering (SERS), with the use of a *p*‐mercaptobenzoic acid reporter molecule, as well as for two photon imaging by direct irradiation with a femtosecond Ti:Sapphire laser setup at 800 nm. It has been shown by several studies that particles with sharp edges (i.e., nanostars) may constitute a superior drug delivery/photothermal platform as surface spikes tend to penetrate cell membranes more efficiently and the particles can be retained into the cytosol for prolonged timeframes (Adnan et al., 2016; Black et al., 2014; Dasgupta et al., 2014; Yan et al., 2015). Similar strategies have been followed with the use of Au nanocages where the NPs can be used both as drug carriers and as imaging probes to allow for image‐guided drug delivery applications. For example, the excellent photothermal properties of Au nanocages can be combined with a phase‐change material that undergoes solid–liquid type of transition within the hyperthermia window to irradiation‐controlled drug release at tumor sites in a spatiotemporally controlled manner (Chen et al., 2019). At the same time, labeling with a radionuclide, Au nanocages allow for precise tracking of their fate and trajectories in vivo for image‐guided cancer therapy (Qiu et al., 2021). Duan et al. elegantly demonstrated the potency of Au nanocages as RNA theranostics and probed the role of size, surface chemistry and charge on tumor penetration properties with the use of orthotopic tumor models (Bao et al., 2017) (Figure 3m–o). miRNA was immobilized electrostatically by coating with cationic polyethylene imine followed by suppression of the excess of positive charge with hyaluronic acid (HA). As in the case of nanostars (see example as discussed above), HA‐passivated nanocages with average size of 30 nm showed the highest tumor accumulation and the deepest tumor penetration 6 h postinjection. Higher retention rates were also observed in orthotopic liver models, again due to passive accumulation by the EPR effect.

An alternative approach to red‐shift the SPR of Au NPs for NIR phototherapies and theranostics is to simply induce controlled aggregation of spherical Au NPs, resulting in interparticle plasmonic coupling (Ghosh & Pal, 2007; Guan et al., 2019; Yuan et al., 2015). Gao et al. reported on a simple Au NP protocol comprising simple mixing of Au NPs with phosphate buffered saline (PBS) or aqueous glucose solutions (M. Sun, Fei, et al., 2016). The rapidly formed Au aggregates showed superior photothermal conversion efficiency (52%) compared to Au nanorods (26%) under the same experimental conditions. Paulus et al. (Nguyen et al., 2021) recently demonstrated that Au nanoaggregates could greatly augment photoacoustic and optical coherence tomography signals in a preclinical rabbit model to visualize neovascularization of the eye. More elaborate approaches have been reported with the use of polymers to induce ordered aggregation in hierarchically more complex architectures (P. Huang et al., 2013; S. Wang et al., 2010). Our group reported on a similar approach with a polymer‐drug conjugate as a macro‐aggregator owing to the intrinsic affinity of the gemcitabine drug molecule to Au surfaces. The resulting hierarchical ensembles had very high drug loading rate (>50%) and could elicit synergistic photothermal and drug‐induced cytotoxicity against pancreatic cancer cells (Joubert & Pasparakis, 2018). These approaches are highly versatile given the synthetic accessibility of Au NPs combined with further surface functionalization strategies via Au—S bond formation or followed by simple aggregation protocols.

Recently, Au NPs of unusual geometries were reported for precision medicine applications that truly push the boundaries of the current status in the biomedical field. For example, P. Huang et al. (2014) reported a novel liquid–liquid–gas synthesis protocol with the aid of ultrasonication to form Au bellflower‐type of nanostructures. The latter showed superior photothermal conversion efficiency (74%) due to the multiple‐branched petals which couple local plasmonic effects; this geometry also resulted in excellent photoacoustic properties, rendering nano‐bellflowers an excellent theranostic probe for simultaneous photothermal therapy and photoacoustic imaging. In another study, He et al. (W. Wang et al., 2019) fabricated polymer‐coated cone‐shaped Au nanoswimmers that can navigate in suspension by ultrasound waves to porate HeLa cell membranes by NIR laser irradiation and deliver molecular cargo in the cytosol; this approach could potentially prove useful in single‐cell therapeutics and diagnostics.

Recently, it was reported that upon light irradiation Au NPs can generate cytotoxic reactive oxygen species at sufficient rates for phototherapeutic applications. The generation of ^1^O_2_, the main component for PDT, by direct irradiation of Au (and Ag) NPs has been demonstrated independently by several research groups (Chadwick et al., 2016; Pasparakis, 2013; Vankayala et al., 2011). ^1^O_2_ is produced by direct interaction of plasmons and hot electrons with molecular oxygen but also there is a complementary photothermal pathway which greatly enhances the overall yield during irradiation with ultrafast laser sources; a distinct advantage of Au NPs is that they display excellent photostability during two‐photon excitation compared to rapidly photobleaching organic photosensitizers (Gao et al., 2014; C. Jiang et al., 2013; T. Zhao et al., 2012). In a series of reports, Hwang et al. reported on Au NP‐mediated PDT with the use of nanoshells (Vankayala, Lin, et al., 2014), and nanorods (Vankayala, Huang et al., 2014), as these nanomaterials can also generate ^1^O_2_ in the NIR regime; the same group expanded the concept and developed densely spiked Au NPs with extinction coefficient in the second NIR window (ca. 870–1100 nm) which could exert direct PDT by laser irradiation (Vijayaraghavan et al., 2014). A similar core‐petal Au nanostructure was developed by Nam et al. (A. Kumar et al., 2014), that could act as dual photothermal‐PDT agent as well as a SERS probe to monitor DNA cell changes postirradiation. These studies constitute a paradigm shift in cancer phototherapeutics as they introduce bimodal therapies; these are photothermal and photodynamic modalities that could be performed under a single‐nanomaterial platform.

## 3 AG NPs: ANTIVIRAL, ANTIBACTERIAL, AND CANCER THERAPEUTICS

**FIGURE 4:**
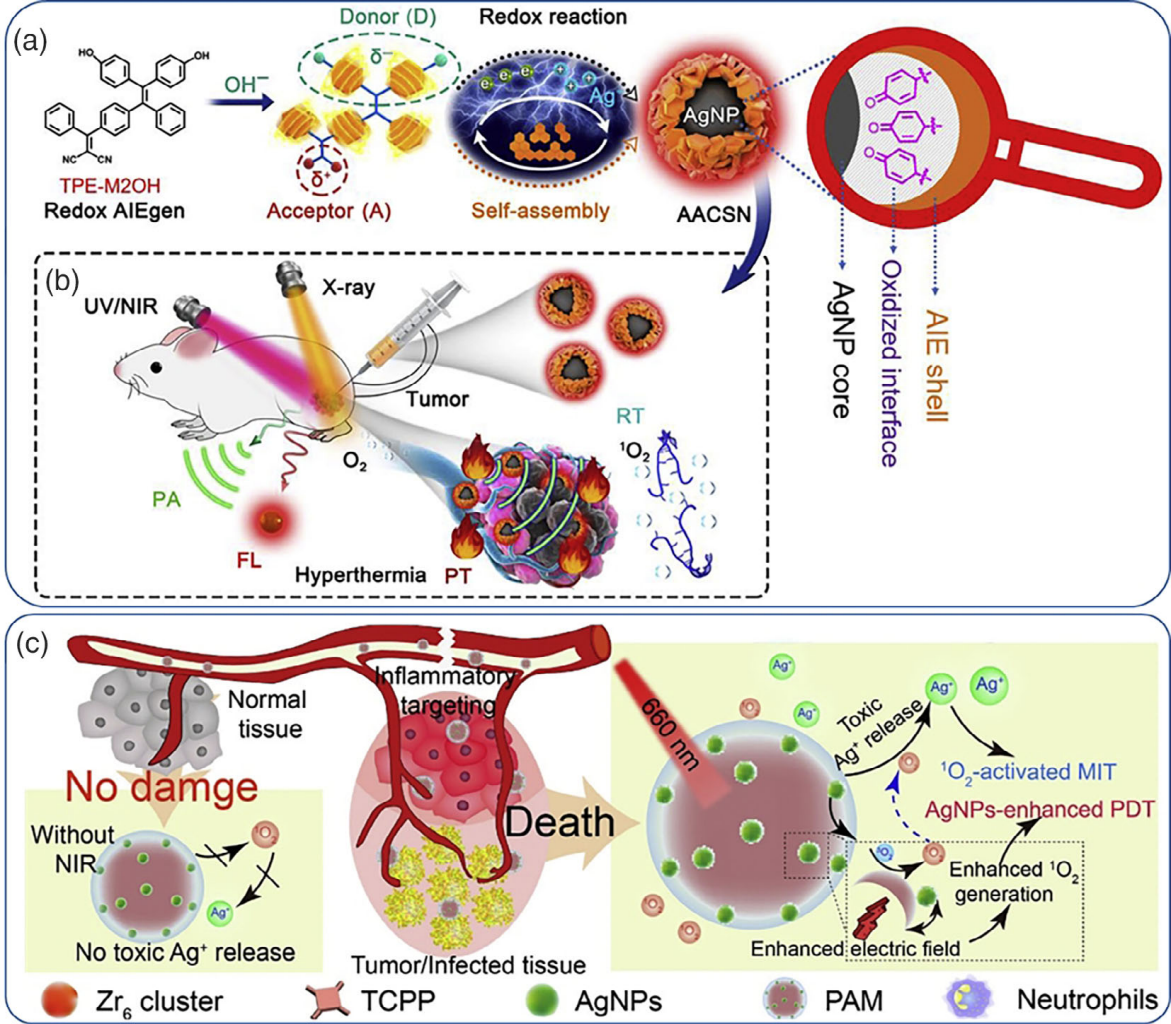
Schematic of the construction of Ag nanoparticle (NP)‐based multifunctional nanoformulation with redox reaction between Ag^+^ and AIEgen in a single‐nanoparticulate form in (a) allowing for multimodal image‐guided tumor treatment in (b). Inflammation targeting and synergistic effects of Ag NPs with porphyrinic porous coordination network for photodynamic therapy (PDT) generated ^1^O_2_ and Ag^+^ release and targeted metal‐ion therapy (MIT) in (c). Copyright by Elsevier (X. He, Peng, et al., 2020; L. Zhang et al., 2020)

Ag in its nanoparticulate form is by far the most widely used metal in the research of antibacterial and antiviral nanoformulations (Sánchez‐López et al., 2020; X.‐F. Zhang et al., 2016). Ag NPs of 10 nm seem to effectively inhibit extracellular SARS‐CoV‐2 (Jeremiah et al., 2020) by direct interaction with the virion capsids and have been proposed as a potential addition to the SARS‐CoV‐2 (Allawadhi et al., 2021) arsenal; a clinical study showed that ARGOVIT (Ag NPs rinse solution) could prevent SARS‐CoV‐2 contagion among health workers with an efficiency of 84.8% (Almanza‐Reyes et al., 2021) while recently, it was shown that Ag NPs have strong immunomodulatory response as vaccine adjuvants against influenza virus (Sanchez‐Guzman et al., 2019). Ag NPs are also highly toxic against both gram‐positive and gram‐negative bacteria via multiple mechanisms and mitigate the issue of drug resistance development as encountered with conventional antibiotics owing to their mechanism of action (Le Ouay & Stellacci, 2015). The proposed antibacterial mechanism of Ag NPs comprises the release of Ag^+^ ions that disrupt the negatively charged cell wall inducing cell lysis; Ag^+^ ions are believed to block DNA replication and the functioning of certain respiratory enzymes via thiol‐coupling interactions. Ag NPs also disrupt the mitochondrial respiratory chain and interrupt ATP synthesis. Finally, Ag NPs generate ROS which, above a certain concentration threshold, lead to cell apoptosis. These mechanisms have also been observed with mammalian cells and these attribute for the observed hemolytic properties of Ag NPs (Wijnhoven et al., 2009). The systemic toxicity of orally or intravenously administered Ag NPs has been unambiguously proven (de Jong et al., 2013; Van Der Zande et al., 2012), and it has been shown that the toxicity profile is correlated to the amount of Ag^+^ released during exposure. Particle surface modification strategies or the combination with other metals are possible means to somewhat mitigate the issue of systemic toxicity (for example see the next section on bimetallic Au–Ag hybrids); nevertheless there are several reports on the Ag^+^ release properties of Ag NPs to formulate nanomedicines not only for antibacterials' design but also for multimodal cancer therapy; Tang et al. coated Ag NPs with aggregation‐induced emission luminogens (AIEgen) forming a NIR absorbing formulation that was shown to have potent antitumor efficacy in vivo with simultaneous imaging capabilities owing to the X‐ray sensitization properties of the Ag metal core (Figure 4a,b) (X. He, Peng, et al., 2020). Zhang et al. designed a nanoformulation with tunable Ag^+^ release triggered by NIR irradiation. Ag NPs were decorated on the surface of porphyrinic porous coordination network (Figure 4c); the latter is a well‐known photosensitizer that generates cytotoxic ^1^O_2_ upon NIR irradiation. The combination with Ag NPs was twofold: (1) to enhance the ^1^O_2_ generation efficiency due to local electric effects and (2) to trigger on‐demand Ag^+^ release confined at the area of irradiation. The proposed formulation was tested both for its antitumor properties against CT26 tumor bearing mice as well as for its bactericidal properties against *Staphylococcus aureus* (L. Zhang et al., 2020). In a similar context, Gu et al. used Ag NPs to enhance the generation of ROS which could in turn lead to augmented autophagy‐driven tumoricidal effects upon irradiation (Wu et al., 2016). The ROS regulating properties of Ag NPs via stimulated Ag^+^ release have also been used for the treatment of rheumatoid arthritis which causes severe inflammation by an auto‐immune response mechanism (Rao, Aziz, et al., 2018; Rao, Roome, et al., 2018; Y Yang, Yi, et al., 2021). Interesting efforts to enhance the irradiation‐induced formation of ROS (and primarily ^1^O_2_) have focused on the synthesis of Ag NPs with anisotropic geometries (MacIa et al., 2019) with enhanced local electric fields superior to their spherical counterparts (Planas et al., 2016), which in turn enhance the ^1^O_2_ output from confined photosensitizers. These approaches are highly appealing in that they show the remarkable therapeutic potency of Ag NPs at the tumor/inflamed sites; however, the systemic toxicity is a persistent issue that still hampers the clinical translation in precision medicine applications.

**FIGURE 5:**
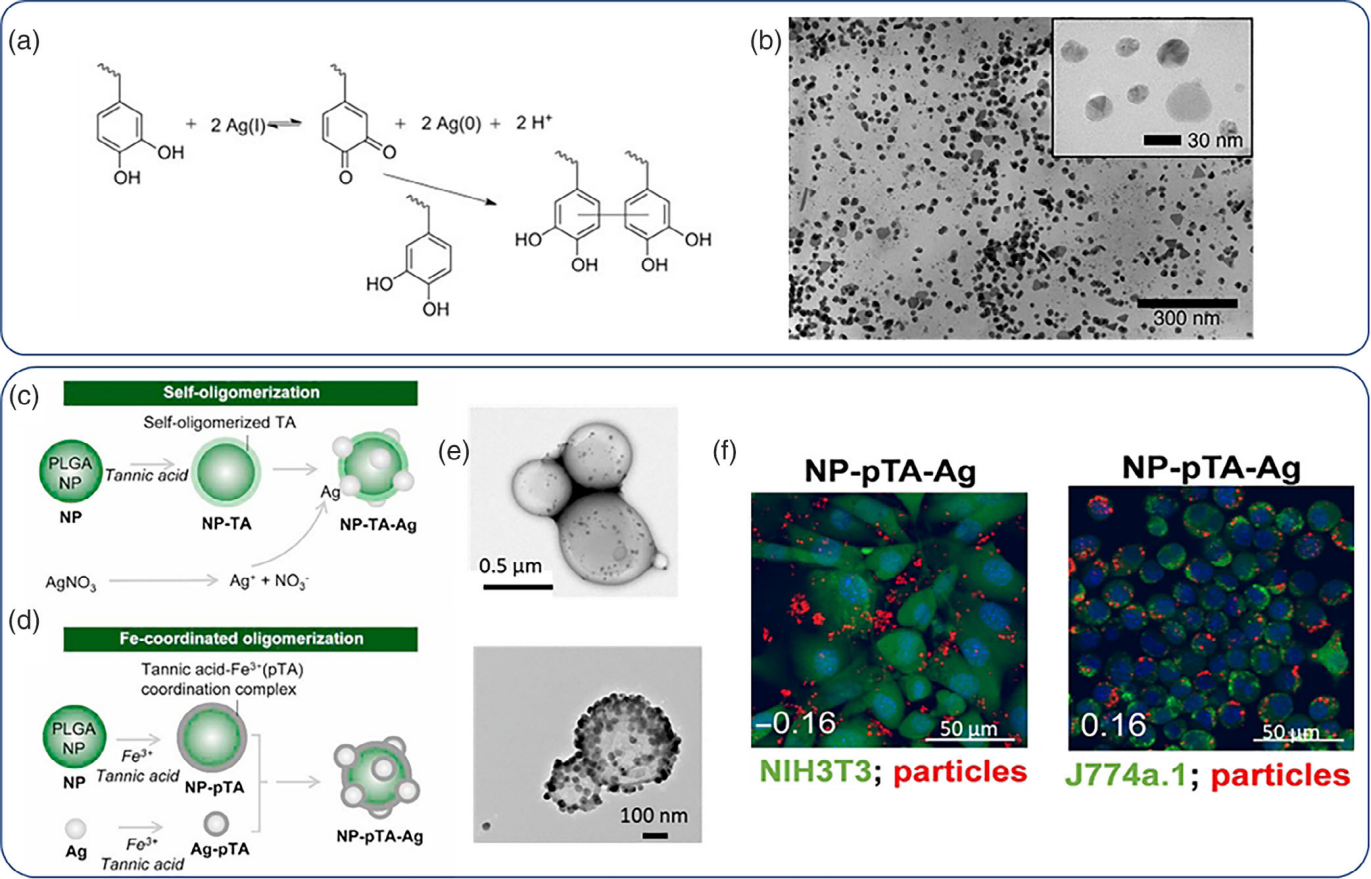
Catechol induced in situ reduction of Ag(I) to form Ag NPs in (a) and Ag NPs as produced by this method in (b). Ag NP embossed PLGA NPs by tannic acid reduction and Fe^+^ coordination in (c) and (d), and their respective TEM images in (e). Selective higher uptake of the NPs by J77a.1 macrophages compared to NIH3T3 fibroblast cells (NPs with red signal in (f)). Copyright by Elsevier (Elnaggar et al., 2020; Fullenkamp et al., 2012)

This partly explains why Ag NPs are preferably used as fillers in biomaterials design to prevent topical bacterial infection or as additives in surface coatings to exert bactericidal activity. To this end, Ag NPs have been used as coating additives in films and membranes (L. Yu et al., 2019), textiles (Noor et al., 2019), and as additives in sprayable formulations (Trabucco et al., 2021). Of great importance is the NP coating of urinary catheters which are prone to bacterial infections and pose pressures on healthcare systems (Knetsch & Koole, 2011). Vasilev et al. proposed a simple Ag NP immobilization strategy comprising Ag NPs coated with marcaptosuccinic acid to prevent rapid metal oxidation and improve the surface immobilization properties. Then plasma polymerization of allylamine was performed on a substrate affording a thin polycationic film due to the abundance of primary amine groups which facilitated efficient NP adhesion; this simple strategy proved to be highly bactericidal against clinical relevant pathogens, namely, *Staphylococcus epidermidis*, *S. aureus*, and *Pseudomonas aeruginosa* (Taheri et al., 2014). Another simple and potentially scalable approach to decorate urinary catheters was proposed by Yassin et al.; first, an initial polydopamine (PDA) coating was introduced on the catheter's surface by simple dipping in alkaline dopamine solutions followed by in situ reduction of AgNO_3_ (Black et al., 2011) to form Ag NPs coordinated at the catechol moieties of the polydopamine layer. The coated catheters showed remarkable bactericidal activity against *S. aureus* and *Escherichia coli* which was attributed to cell membrane lysis primarily by Ag^+^ disruption (Yassin et al., 2019). A variation of this approach was used by Neoh et al. (R. Wang et al., 2015) who modified silicon catheters with multiple PDA/Ag NP layers by sequential dip coating followed by a final coating step with an antifouling poly(sulfobetaine methacrylate‐co‐acrylamide polymer); the modified catheters showed excellent antibacterial activities and resisted encrustation by up to 45 days, thus outperforming commercially available Ag NP‐coated catheters (Dover™). Direct catechol reduction by Ag(I) followed by simultaneous polymer crosslinking and in situ formation of Ag NPs was reported by Messersmith et al. (Fullenkamp et al., 2012) who developed this simple method to construct antibacterial hydrogels (Figure 5a,b). The proposed method is quite simple as it combines (synthetically accessible) catechol‐terminated branched and linear PEG polymers with AgNO_3_ by simple mixing in alkaline buffers. The hydrogels produced are highly hydrophilic due to the PEG content and are impregnated by the in situ formed AgNPs which interestingly were shown to release their Ag content in a sought‐after zero‐order rate. The hydrogels were found to be antibacterial but without compromising mammalian cell viability due to the relatively low Ag overall content. This hydrogel formation protocol with simultaneous Ag NP formation has been adopted and refined by other research groups with the use of functional polymers for wound healing and antibacterial coating applications (Ghavaminejad et al., 2016; Pham et al., 2020) owing to the catechol‐driven Ag NP formation simply by incorporating self‐polymerizable dopamine derivatives. Yeo et al. used tannic acid coating (either in the form of oligomers or as coordinated oligomerized network by Fe^3+^ on poly(lactic‐co‐glycolic acid) PLGA NPs to reduce Ag^+^ to metallic Ag NPs on the surface of the NPs (Figure 5c–f) (Elnaggar et al., 2020). This approach allowed for efficient Ag NP decoration of PLGA NPs which were additionally loaded with pexiganan, a broad spectrum antibacterial. The formulation could be uptaken selectively by macrophages and not by non‐phagocytic cells (i.e., fibroblasts), thus mitigating the systemic toxicity issue of Ag NPs rendering the system suitable for intracellular bacterial infections. Other methods to homogeneously impregnate Ag NPs to polymer matrices include direct reduction of AgNO_3_ (i.e., by NaBH_4_, Niu et al., 2018) by other natural extracts as reducing agents (Jadhav et al., 2016; Martínez‐Higuera et al., 2021), peptides (Jo et al., 2014)), UV irradiation (Henríquez et al., 2014), direct blending of polymer matrices with sterically protected preformed Ag NPs (Jahan et al., 2019; Rolim et al., 2019) or via robust electrochemical (Nešović & Mišković‐Stanković, 2020) and even electrohydrodynamic routes (J. Yang et al., 2020).

## 4 BIMETALLIC HYBRID AU–AG NP SYSTEMS

**FIGURE 6:**
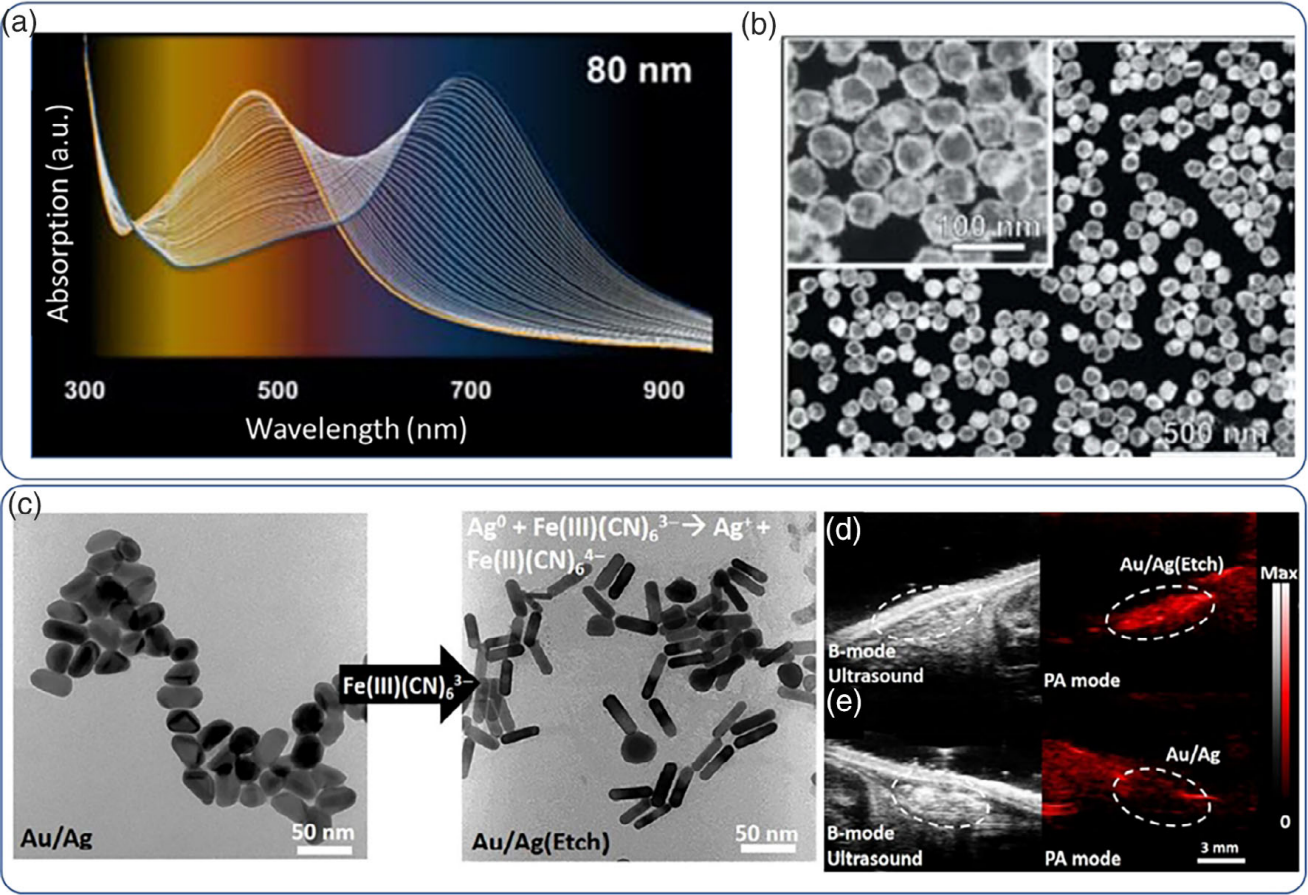
Time‐resolved evolution of the absorption spectrum of hollow Au–Ag nanoshells with characteristic red‐shifting of the surface plasmon resonance (SPR) band in (a), high‐angle annular dark‐field scanning TEM image of hollow Au–Ag 60‐nm nanoshells with thin shells and large voids in (b). TEM image of Ag‐coated Au nanorods NRs and stripping off of the Ag coating upon exposure of a mild etchant in (c), photoacoustic contrast signal recovery (red signal) upon Ag etching tracked by ultrasound imaging of subcutaneously injected Au–Ag nanorods on a mouse model in (d) and (e). Copyright by American Chemical Society (Kim et al., 2018; Russo et al., 2018)

Au and Ag have similar SPR bands and lattice constants and can be reduced under nearly identical conditions (Liz‐Marzán, 2005). To obtain spherical bimetallic NPs, there are two main methods, top‐down, for example, by ablating a bulk alloy sample to obtain NP fragments, and bottom‐up, by using suitable chemistries to obtain noble metallic NPs from the corresponding metal ions (i.e., by reducing Ag^+^ or Au^3+^) (Loza et al., 2020). The latter method is more commonly used as it is simpler, accessible from most laboratories and allows for formation of nonspherical NP geometries. Simultaneous reduction of metal precursors can lead to alloyed Au/Ag NPs while sequential reduction can lead to the formation of core‐shell type geometries with the more noble metal (Au) occupying the core and the less noble Ag forming the shell (Blommaerts et al., 2019). The reverse reduction sequence (first Ag followed by Au) is possible (Kumar‐Krishnan et al., 2017) but can often lead to dissolution of preformed Ag NPs by Au ions and end up forming interesting shapes (i.e., hollow particles, Russo et al., 2018; Figure 6a,b). Alloyed Au/Ag NPs display a single‐shifted SPR ranging between pure Au and Ag, depending linearly on the stoichiometry of the two metals. For spherical core‐shell NPs two separate plasmon bands are usually observed. Collective optical properties can also be achieved by controlled Au/Ag particle confinement which allows for enhanced plasmon coupling via the so‐called core‐satellite topology (I. Choi et al., 2012). Alternative biosynthetic methods to form bimetallic Au/Ag NPs comprise the use of *E. coli* first to form Au NPs, followed by reduction of Ag ions (X. Jiang et al., 2020; Y. Yang et al., 2022). Bimetallic NPs have combined properties of their constituent metals and hence allow for multiple functionalities under a single‐nanoparticulate platform. For example, Hu and co‐workers reported core‐shell Au–Ag NPs to harness the X‐ray contrast enhancement of Au and the antibacterial activity of Ag to devise a X‐ray trackable nanomedicine against methicillin‐resistant *S. aureus* (MRSA) (Huo et al., 2014). A similar approach was employed by Jokerst et al.; however, their proposed formulation comprised nanorod‐shaped Au–Ag hybrids NPs (Kim et al., 2018); Au nanorods were first synthesized which were subsequently coated with a Ag layer which masked the longitudinal plasmon band; triggered release of Ag^+^ was activated by addition of a mild oxidant (ferricyanide ions [Fe(CN)_6_]^3−^) that gradually etched the Ag shell and restored the Au plasmon band enabling for simultaneous NIR photoacoustic imaging of the bactericidal activity against MRSA (Figure 6c–e). A similar formulation again comprising Au–Ag nanorods was employed by Sheng et al. (Mei et al., 2020) albeit the researchers exploited the NIR‐II optical window (laser irradiation at 1064 nm) to activate the NPs. Zhou et al. (J. He, Qiao, et al., 2020) proposed an hyaluronate gel impregnated with Au–Ag nanoshell with multiple functionalities, suitable for wound healing from severe bacterial infections. The nanoshells exhibited red‐shifted plasmon band owing to the Au content which can elicit photothermal effects by NIR laser irradiation while Ag served as the Ag^+^ reservoir further enhancing the bactericidal efficiency. In addition, the researchers explored metallic NPs as SERS probes to continuously monitor bacterial populations during treatment even at low CFUs (300 CFU/ml) up to 8 days. The synergistic photothermal/Ag^+^ effects resulted in significantly faster wound healing timeframes compared to control subjects. Yang et al. (Ding et al., 2017) exploited the large two‐photon luminescence signal of hybrid Au/Ag NPs to image MRSA via electrostatic attachment of the positively charged NPs to the bacterial walls; crowding of the NPs on the bacterial walls resulted in confined aggregation which amplified the signal output and by irradiation with a pulsed femtosecond laser it was possible to induce photothermal/ROS‐mediated bactericidal activity. The synergism of photothermal treatment with cytotoxic Ag^+^ release was also studied by Zhou et al. who synthesized hollow Au–Ag nanoshells for color cancer therapy; the metallic composition allowed for the installation of SERS probes for image‐guided photothermal therapy (J. He et al., 2021). Star‐shaped Au–Ag bimetallic NPs with red/NIR SPR have also been reported for photothermal therapeutics; Liu et al. proposed (Cheng et al., 2012) a simple synthesis protocol to form spiky nanostars by simultaneous reduction of HAuCl_4_ and AgNO_3_ followed by polymer coating. It was possible to control the number and morphology of the Ag spikes on the surface of the NPs simply by adjusting the Au:Ag ratio and in turn tune the optical properties. The proposed bimetallic NPs exhibited selective cytotoxicity in vitro by laser irradiation at 800 nm.

## 5 AU‐ AND AG‐BASED BIOENSORS

**FIGURE 7:**
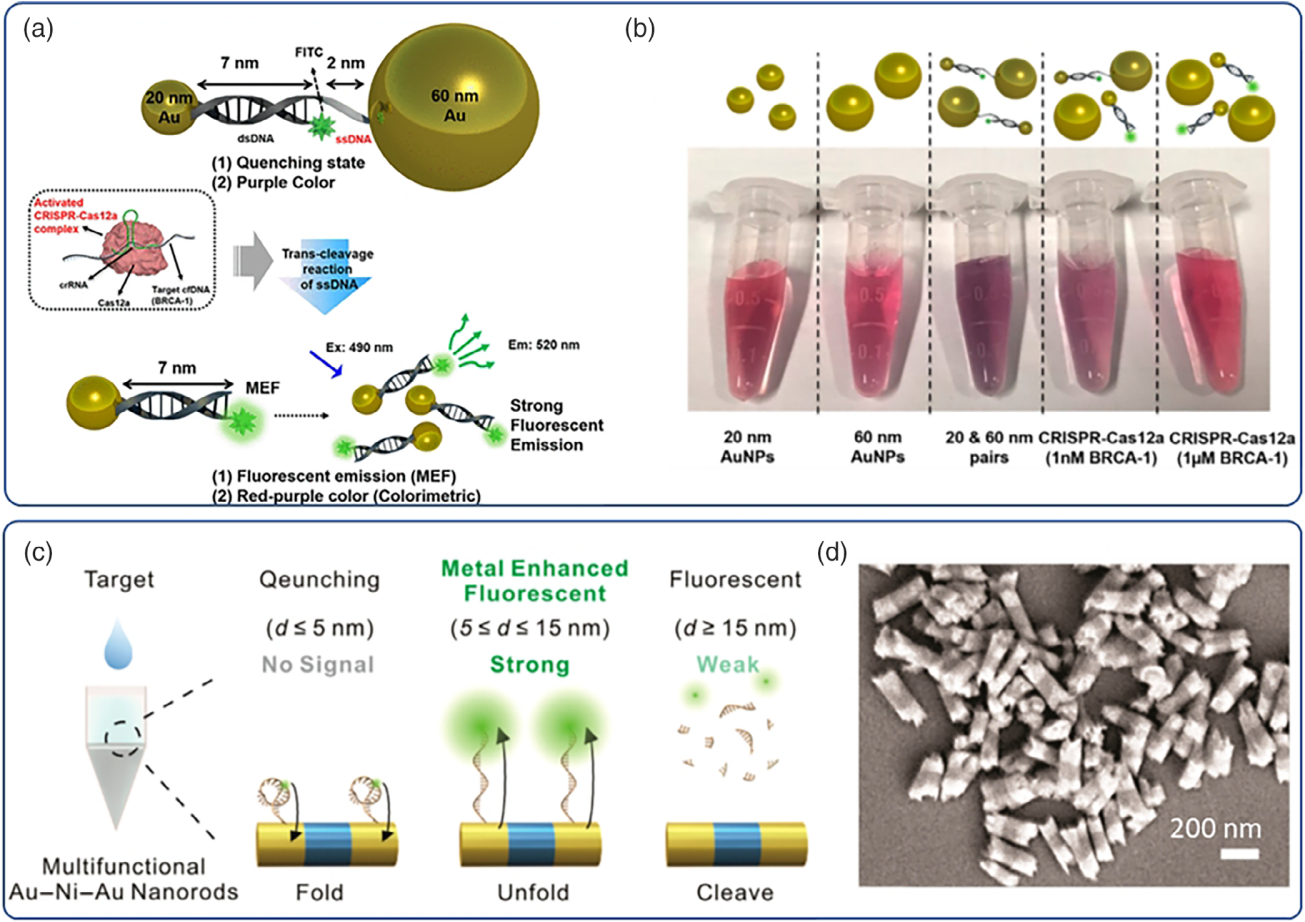
CRISPR induced MEF with Au NPs; 20 nm Au NPs are coupled with 60 Au NPz via a FITC‐containing double‐stranded DNA (reporter molecule) and a single‐stranded DNA molecule (quenched OFF state). Dequenching (ON state) is achieved by CRISPR‐*Cas12a* activation via crRNA and target cfDNA by trans‐cleavage effects in (a), naked eye visible color changes of the assay at various stages with characteristic changes upon NP pair formation (purple hue) and dequenching (restoration and MEF signal) events by CRISPR in (b). Schematic showing the characteristic MEF quenching and dequenching of the sensor based on the proximal distance changes between the reporter molecule (5(6)‐carboxyfluorescein as molecular beacon) and the metallic center (Au–Ni–Au rods) in respect to folding/unfolding events of DNA strands in (c), and typical scanning electron microscopy (SEM) image of the magneto‐plasmonic sensors in (d). Copyright by American Chemical Society (J.‐H. Choi et al., 2021; Lee et al., 2019). CRISPR, clustered regularly interspaced short palindromic repeats; MEF, metal‐enhanced fluorescence; NPs, nanoparticles

Au/Ag NPs are the most commonly used nanoparticulate platforms for biosensing applications broadly based on direct colorimetric (aggregation‐based) visualization (Vilela et al., 2012), metal‐enhanced fluorescence (MEF) (Y. Jeong et al., 2018) and surface‐SERS (Langer et al., 2020); Au NPs are also commonly used in colorimetric detection technologies (i.e., lateral flow immunochromatic assays) due to their SPR in the visible regime (Alberti et al., 2021; Sabela et al., 2017). These methods are often integrated under a single‐biosensing platform to augment signal sensitivity and improve device utility. The potency of Au/Ag NPs in pathogen detection was manifested during the 2019 SARS‐Cov‐2 pandemic where many research groups presented robust biosensing platforms to mitigate the pandemic crisis; there are currently numerous commercially available platforms to detect SARS‐CoV‐2 which have been reviewed in more detail elsewhere (Hsiao et al., 2021; Hsieh et al., 2021). For example, Jiao et al. (C. Huang et al., 2020) successfully developed a classic IgM antibody colorimetric detection assay for SARS‐Cov‐2 based on a lateral flow assay with Au NPs which showed excellent sensitivity and specificity (100% and 93%, respectively). It is noteworthy that the widely used concept of immobilizing detection molecules or signal probes (i.e., fluorescent dyes) on the surface of metallic NPs has been greatly boosted by the integration of aptamers as surface modifiers which exert tremendous diversity toward multifarious targets (Sharifi et al., 2020). Nucleic acids can also serve not only as aptamers but also as pathogen NP aggregators for direct colorimetric detection. Teengam et al. (2017) developed simple paper‐based sensors based on peptide nucleic acids (a oligo‐peptide DNA mimic with improved chemical stability) that could induce Ag NP aggregation followed by colorimetric detection; the proposed design could be tested for different pathogens (Middle east respiratory syndrome coronavirus (MERS‐CoV), Mycobacterium tuberculosis (MTB), and Human papillomavirus (HPV)) with adequate limit of detection. Xu et al. (2021) proposed an interesting strategy to further improve the detection limit of colorimetric sensors and designed a hybrid probe based on Au NPs conjugated with M13 phage; the probe could capture H1N1 via antibody–antigen interactions followed by magnetic separation; virus containing complexes were subsequently hosted in bacterial cells developing a naked‐eye detectable signal owing to the LacZa gene in the M13 genome. The limit of detection was as low as 50 PFU/ml, far more potent than quantitative polymerase chain reaction (PCR) methods. Potentially more accessible methods to integrate signal amplification mechanisms for direct colorimetric detection are based on gene editing methods (i.e., CRISPR, clustered regularly interspaced short palindromic repeats; Kaminski et al., 2021) as they harness nucleic acids both as recognition elements and as amplification mechanisms and can be easily mounted on the surface of metallic NPs mostly via Au/Ag‐S bonding (van Dongen et al., 2020). More importantly the *Cas*‐enzymes have proven selectivity and sensitivity down to zeptomolar (10^−21^ M) concentrations rendering them excellent transducer/signal motifs. Fu et al. (2021) systematically analyzed the effect of tethering *Cas9* (i.e., *LbCas12a* and *AsCas12a*) substrates on SNAs, on the enzyme trans‐cleavage activity of the enzymes; DNA strand density and length were found to be critical factors on the enzyme activity and by design optimization they could develop a SNA/CRISPR diagnostic for HPV‐16 detection with low detection limit down to picomolar range. Cao et al. (2021) modified reverse transcription loop‐mediated isothermal amplification with Au NPs as CRISPR‐Dx platform by utilizing nucleic acid hairpin transducers to induce Au NP aggregation upon recognition of N and E genes of SARS‐CoV‐2, which could be directly visualized within 45 min. In a similar context, Abnous et al. (2021) developed a CRISPR‐Au NP sensor ofaflatoxin M1 based on the surface interaction of Au NPs with 4‐nitrophenol (yellow) which leads to the formation of colorless 4‐aminophenol; the switching only takes place upon activation of a CRISPR‐*Cas9* system with an aflatoxin aptamer component. The proposed sensors showed very good sensitivity down to nanomolar concentrations. In another study, J.‐H. Choi et al. (2021) conceptualized the coupling of a CRISPR‐*Cas12a* with MEF (Figure 7a,b); MEF is highly dependent on the distance from the surface of Au NPs in that metallic NPs can act as fluorescence quenchers in close proximity to the surface of the NP while MEF takes place at longer distances (i.e., >2 nm); therefore Au NPs dimers linked with a ssDNA‐fluorescent tag were synthesized which could switch from an OFF (quenched) state to a ON signal (MEF) upon CRISPR Au NP dissociation; the sensor could rapidly detect the breast cancer gene‐1 (BRCA‐1, an important prognostic biomarker for breast cancer) at picomolar concentrations. MEF was also elegantly exploited by Lee et al. (2019) who reported on novel magnetoplasmonic nanorods (with the structure Au–Ni–Au) with orthogonal magnetic and optical properties (Figure 7c,d); these hybrids could be used as immunomagnetic extractors owing to their Ni content in order to isolate exosomes, while the Au components were designed to detect specific miRNA sequences by immobilization of suitable molecular beacons that induced quenching/de‐quenching (MEF signal) transition via hybridization reactions. An elegant approach toward quantitative detection of SARS‐CoV‐2 was reported by Alafeef et al. (2020) employing conductive graphene substrates bearing Au NPs with antisense ssDNA oligos targeting the N‐gene of the virus. The proposed electrochemical sensor could detect SARS‐CoV‐2 RNA down to 6.9 copies/μl and could also distinguish signal from similar RNA analytes such as SARS‐CoV, MERS‐CoV, or even negative COVID‐19 samples from healthy subjects. Both Au and Ag NPs serve as excellent metallic substrates for plasmon‐enhanced Raman scattering (PERS). PERS (or SERS, i.e., SurfaceERS) can be performed in solution or on surfaces and metallic NPs can enhance the signal output by up to × 10^14^ (X. Wang, Huang, et al., 2020) allowing for reliable sing‐molecule detection of nanoscale spatial resolution with immediate interest in the field of biosensors for disease diagnosis and therapeutics monitoring, food safety, bio‐security applications (Eom et al., 2017; Deng et al., 2019; Jeon et al., 2020; K. Wang, Sun, et al., 2020; Ma et al., 2020), and more recently for the detection of SARS‐CoV‐2 (H. Liu et al., 2021). Ag NPs are more efficient SERS enhancers, however, they are less stable, more difficult to functionalize (due to the weaker Ag‐S bond compared to Au‐S), and more difficult to control their sizes (X. Wang, Huang, et al., 2020).

**FIGURE 8:**
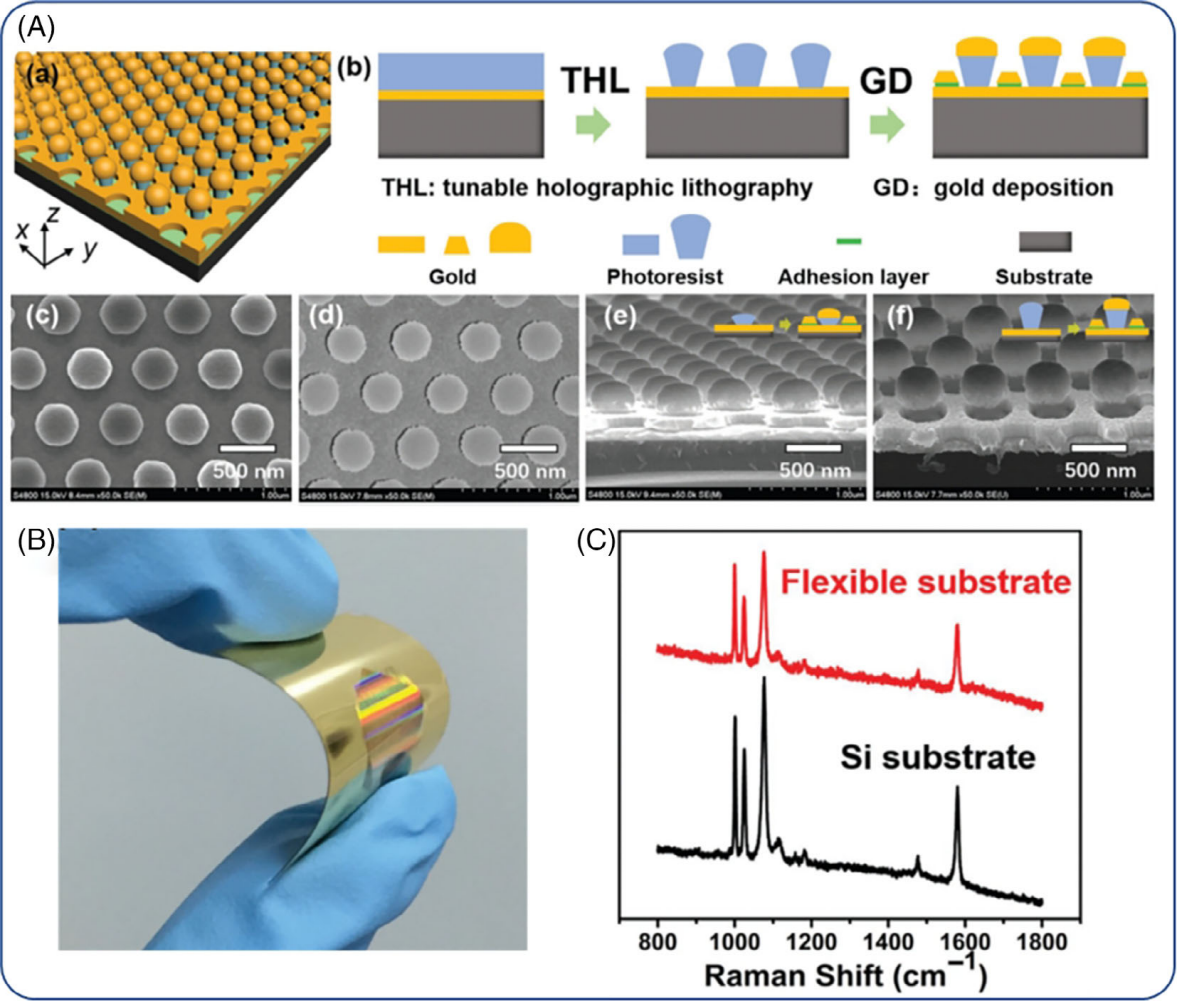
Fabrication procedure of Si‐patterned surfaces for surface‐enhanced Raman scattering (SERS) in (A) (a,b), SEM images of top and side views of the surfaces (c–f); characteristic bending of the patterned nanostructures on a polyethylene substrate in (B) and typical SERS spectra of flexible and Si patterned surfaces in (C). Copyright by Wiley (B. Liu et al., 2018)

Ag and Au NPs are commonly used for the fabrication of SERS sensors in solution and at surfaces (Mosier‐Boss, 2017). There are two, often complementary, preconditions for both approaches to enhance signal output: (1) to maximize the so‐called hot spots among NPs populations and (2) prolongation of the time of interaction of the target molecules with the SERS substrate (Li et al., 2003; X. Wang, Sun, et al., 2020). In recent years, lithographic methods have been employed for the construction highly ordered Ag/Au substrates for SERS applications which fully harness the spatial requirements for optimum signal detection (Figure 8) even with the use of flexible substrates allowing for further exploitation for in situ monitoring applications, for example, in wearable devices and soft robotics (B. Liu et al., 2018).

In recent years, DNA origami has emerged as a powerful means to immobilize NPs for SERS applications robustly and in a more cost‐efficient manner compared to lithographic techniques (Dey et al., 2021; Hong et al., 2017). DNA origami constitutes a method where DNA can be arranged into higher‐ordered structures via preprogrammed hybridization of DNA strands; the method can be facilely combined with Au and Ag NPs owing to the accessibility of Au–NP hybrids via Au–thiol (and the less stable Ag‐S) bond formation. Keyser et al. (Thacker et al., 2014) demonstrated efficient immobilization of Au NPs on DNA origami nanosheets which enabled the formation of NP dimers with sub‐5 nm gap sizes via DNA hybridization anchoring for efficient SERS signaling. Finkelstein et al. (Pilo‐Pais et al., 2014) further extended this approach to utilize tetrameric Au NPs ensembles for SERS. In a similar context, Lohmuller and co‐workers (Simoncelli et al., 2016) reported on the optothermal control of the SERS signal output extracted from an Au NP dimer with a temperature sensitive DNA origami template which could reduce interparticle gaps down to 1–2 nm. In an elegant study, Bald et al. (Tapio et al., 2021) introduced DNA origami nanofork antennas sandwiched by Au (or Ag) dimer NPs; the researchers designed hybridization strategies to stabilize the structures and demonstrated excellent electric signal enhancement up to ×10^11^ fold enhancement. Interestingly, these nanodevices could host larger molecules within the hot‐spot cavity allowing for adequate signal detection as was shown with the use of the model protein horseradish peroxidase. The same group reported(Heck et al., 2018) a similar strategy on Ag–DNA origami hybrid nanolenses for protein detection (i.e., streptavidin in this study); the study, probed technical issues that still somewhat hamper the experimental performance of Ag NPs nanolenses compared to theoretical expectations (and to Au counterparts; Heck et al., 2017), which poses future technical challenges for further refinement of Ag NPs coating protocols and immobilization chemistries. Similar studies have emerged with the use of anisotropic NPs such as (the most commonly used) Au nanorods (T. Zhang et al., 2015), and nanostars (Tanwar et al., 2017) as it has been demonstrated that these geometries dramatically enhance the local electric field (Solís et al., 2017).

## 6 CONCLUSION AND FUTURE OUTLOOK

Au and Ag NPs constitute an excellent platform of nanomaterials for multifarious applications in the biomedical context both in basic and applied research as well as in practical applications. Testament of their utility is their rapid adoption as diagnostic platforms for the diagnosis and detection of SARS‐CoV‐2; arguably these nanomaterials have proven to be irreplaceable tools in our arsenal against the pandemic. It is expected that the progress toward more refined diagnostic tools, especially based on the plasmonic properties of Au and Ag, will continue to expand. Plasmon‐based diagnostics constitute a powerful means to detect pathogens and diagnostic biomarkers at minute quantities and the field is constantly fueled by our better understanding on how to control nanoscale geometries with high fidelity that gives rise to new optical properties. On the front of therapeutics, Au NPs have been exhaustively explored in precision medicine and photothermal therapeutics but without proportional clinical translation outcome. The same trend is observed for Ag NPs; however, the latter seem to find translational pathways in antibacterial therapeutics and wound healing as the pharmacological prerequisites are relatively more tolerable (i.e., compared to systemically administered nanoformulations). It remains to be seen whether our growing knowledge on the interactions of these remarkable nanomaterials with human tissue will accelerate their clinical translation. Finally, bimetallic NPs is still a relatively unexplored area which is rapidly expanding and may lead to novel applications that require confined and collective properties from both metals aiming to tackle unmet therapeutic and diagnostic needs.

## AUTHOR CONTRIBUTIONS

**George Pasparakis:** Conceptualization (lead); formal analysis (lead); funding acquisition (lead); methodology (lead); project administration (lead); writing – original draft (lead); writing – review & editing (lead).

## CONFLICT OF INTEREST

The author has declared no conflicts of interest for this article.

## RELATED WIREs ARTICLES

Gold nanoparticle‐mediated photothermal therapy: applications and opportunities for multimodal cancer treatment

## Data Availability

Data derived from public domain resources
